# Dietary niacin Intake and its association with all-cause and cardiovascular mortality rates in individuals with metabolic syndrome

**DOI:** 10.1186/s12937-024-00993-7

**Published:** 2024-08-10

**Authors:** Yuqing Fu, Cong Xu, Guifu Wu

**Affiliations:** Department of Cardiology, The Eighth Affiliated Hospital of Sun Yat-sen University, Shenzhen, 518000 Guangdong China

**Keywords:** Metabolic syndrome, NHANES, Cardiovascular mortality

## Abstract

**Background:**

Individuals with metabolic syndrome face elevated cardiovascular and mortality risks, and there is ongoing debate regarding the cardiovascular effects of niacin and its impact on the prognosis of metabolic syndrome.

**Exposure:**

Levels of dietary niacin intake based on 24-hour dietary recall.

**Methods:**

Kaplan-Meier survival curves were used to compare survival status among quartiles of dietary niacin intake. Weighted Cox proportional hazards models and restricted cubic splines were used to estimate hazard ratios (HRs) and 95% confidence intervals (CIs) for the risk of all-cause and CVD mortality associated with the exposure.

**Results:**

This cohort study included 8,744 participants, and during a median follow-up period of 106 months, 1,552 (17.7%) deaths were recorded, with 511 attributed to cardiovascular disease. Kaplan-Meier curves comparing quartiles of dietary niacin intake showed significant differences in both all-cause and cardiovascular mortality rates (log-rank *p* < 0.001). In the fully adjusted model, the highest quartile of dietary niacin intake was associated with HRs of 0.68 (95% CI: 0.54, 0.87, *P* = 0.002) for all-cause mortality and 0.63 (95% CI: 0.39, 0.78, *P* < 0.001) for cardiovascular mortality.

**Conclusion:**

The results of this cohort study suggest that higher dietary niacin intake is associated with reduced cardiovascular and all-cause mortality risks in the metabolic syndrome population. Furthermore, there appears to be a dose-response relationship between dietary niacin intake and the risks of all-cause and cardiovascular mortality.

**Supplementary Information:**

The online version contains supplementary material available at 10.1186/s12937-024-00993-7.

## Introduction

Metabolic syndrome (MetS) is a cluster of interrelated and accumulating risk factors for cardiovascular disease (CVD), including abnormal blood glucose, lipid abnormalities, hypertension, and central obesity [1, 2]. The prevalence of metabolic syndrome varies significantly globally [3], with surveys showing that the prevalence in the United States has exceeded 30%, continuously increasing over the past 20 years [4]. Patients with metabolic syndrome face a high risks of all-cause and cardiovascular mortality [5, 6]. Studying how to reduce the mortality risk among individuals with metabolic syndrome is of paramount importance.

Dietary niacin, also known as vitamin B3, is considered beneficial for lowering blood lipids [7–9] and plays a crucial role in cellular metabolism [10]. It can improve endothelial dysfunction [11]. Although niacin aids in lipid management [12–15], its cardiovascular protective effect is not universally recognized. On the contrary, one study found an increased risk of developing hypertension with a daily niacin intake exceeding 15.6 mg/d [16]. Several meta-analyses suggest that niacin lacks significant cardiovascular benefits and may even increase the risk of cardiovascular mortality [17, 18]. A recent study suggests that niacin’s adverse effects on the cardiovascular system are mediated through its metabolites and inflammatory effects [19]. Considering the existence of the complex effects niacin may have on lipids and cardiovascular disease, and the lack of large-sample studies on niacin intake among individuals with metabolic syndrome who face a high risk of cardiovascular mortality, this study utilized the National Health and Nutrition Examination Survey(NHANES) database, representative of the US population, to investigate the long-term effects of niacin intake on all-cause and cardiovascular mortality rates among individuals with metabolic syndrome, which was able to make some informative conclusions about the nutritional intake of this population with metabolic syndrome.

## Materials and methods

### Study design and participants

**Fig. 1:**
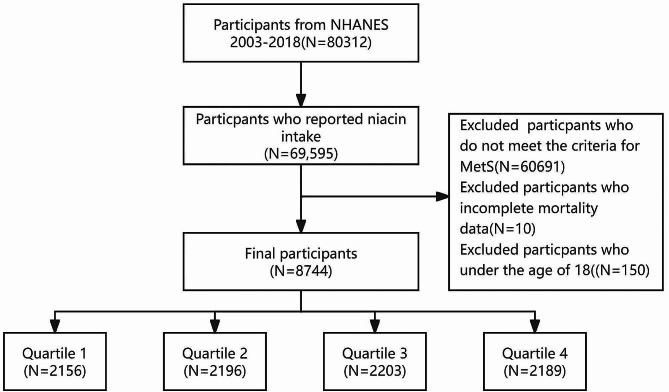
The participants selection flowchart

The data were derived from ten interview cycles of theNHANES, spanning from 2003 to 2018. The NHANES database employs a stratified, multistage probability sampling design to systematically collect health-related data that is representative of the civilian, non-institutionalized US population. The datasets analyzed in this study are accessible at https://www.cdc.gov/nchs/nhanes/index.html [20]. Among the 80,312 participants from the NHANES dataset spanning from 2003 to 2018, a total of 69,595 individuals completed at least one dietary intake interview and reported niacin intake. Among them, 8,904 individuals met the criteria for metabolic syndrome. From these 8,904 subjects, 150 participants under the age of 18 and 10 participants with missing mortality data were excluded, resulting in a final inclusion of 8,744 participants. The participants selection flowchart is shown in Fig. 1. The study was reported according to the Strengthening the Reporting of Observational Studies in Epidemiology (STROBE) reporting guideline.

### Exposure variable

The measurement of dietary niacin intake was conducted through the Dietary Interview component referred to as What We Eat in America (WWEIA), which is a collaboration between the United States Department of Agriculture (USDA) and the Department of Health and Human Services (DHHS). All eligible NHANES participants undergo two 24-hour dietary recall interviews to report the types and amounts of foods consumed in the preceding 24 h (from midnight to midnight). Food energy and 64 nutrients/food components from each food/beverage as calculated using USDA’s Food and Nutrient Database for Dietary Studies. The first recall is conducted face-to-face at the Mobile Examination Center (MEC), while the second recall takes place approximately 3 to 10 days later via telephone interview. The Total Nutrient Intake dataset provides summary records of nutrient intake for each individual. In this study, participants with two niacin intake assessments have their values averaged, while those with only one niacin intake assessment are determined by their single reported value.

### Definition of MetS

The definition of Metabolic Syndrome (MetS) was in accordance with previous guidelines, specifically the National Cholesterol Education Program Adult Treatment Panel III (NCEP ATP III) [21]. Individuals meeting three or more of the following criteria were considered to have MetS: [1] fasting blood glucose (FBG) > 100 mg/dL or undergoing treatment for diabetes mellitus; [2] low levels of high-density lipoprotein cholesterol (HDL-C) (< 50 mg/dL in females, < 40 mg/dL in males) or receiving treatment for reduced HDL-C; [3] plasma triglycerides (TG) > 150 mg/dL or receiving treatment for elevated TG; [4] waist circumference > 88 cm in women or > 102 cm in men; [5] blood pressure > 130/85 mmHg or receiving treatment for hypertension.

### Covariates

Population demographics of the participants were obtained from the NHANES database. Socioeconomic characteristics comprised gender (male or female), age, race/ethnicity (Mexican American, Hispanic, non-Hispanic white, non-Hispanic black, or other race), education level (less than high school, high school, and some college or above), and household income-to-poverty ratio (< 1.0, 1–3, > 3). Additionally, lifestyle habits and comorbidity history were assessed, including smoking status (yes or no), alcohol consumption (yes or no), presence of hypertension (yes or no), diabetes (yes or no), history of cancer, and cardiovascular disease (CVD) (Included self-reported coronary heart disease, stroke, congestive heart failure, myocardial infarction, and heart attack)(Yes or no) and chronic kidney disease(CKD). Furthermore, physical and laboratory examinations such as body mass index (BMI), waist circumference(WC), creatinine, triglyceride(TG), high-density lipoprotein cholesterol(HDL-C), low-density lipoprotein cholesterol(LDL-C), total Cholesterol(TC), alanine aminotransferase (ALT) and aspartate aminotransferase (AST) were considered as potential confounding factors.

### Outcome events

The primary outcomes of this study are all-cause mortality and cardiovascular mortality among individuals with MetS. Mortality data for the follow-up population were obtained from the NHANES public-use linked mortality files as of December 31, 2019. These files are linked to the National Death Index (NDI) through a probabilistic matching algorithm conducted by the National Center for Health Statistics (NCHS). The causes of death were examined using the International Classification of Diseases, 10th Revision (ICD-10), with cardiovascular mortality defined as deaths due to heart disease and cerebrovascular disease, which included the following disease codes: I00-I09 (acute rheumatic fever and chronic rheumatic heart diseases), I11 (hypertensive heart disease), I13 (hypertensive heart and renal disease), I20-I25 (ischemic heart diseases), I26-I28 (pulmonary embolism and other acute pulmonary heart diseases), I29 (various cardiovascular diseases caused by different reasons), I30-I51 (other forms of heart disease), and I60-I69 (cerebrovascular diseases). The follow-up duration was calculated from the initial interview date to the date of patient death or December 31, 2019.

### Statistical analysis

Variables with a normal distribution are presented as mean (standard deviation), whereas variables with a non-normal distribution are presented as median (interquartile range). Categorical variables are expressed as numbers (percentages, %). The Kruskal-Wallis test is used to compare non-normally distributed continuous variables, while the chi-square test is used to compare categorical variables with continuous variables.In this study, all analyses accounted for the complex sampling design of NHANES. According to NHANES guidelines, individuals with two dietary intake data points were assigned the average dietary weight, while those with only one dietary intake data point were assigned the weight from the first intake. Stratification and primary sampling units were also included to ensure accurate representation of the population. For categorical variables, data were presented as unweighted frequencies (weighted percentages), while for continuous variables, data were presented as median (interquartile range).

Kaplan-Meier analysis was utilized to compare mortality rate differences among quartiles of niacin intake, and a weighted Cox regression model was employed to determine hazard ratios (HR) and 95% confidence intervals (CI) to examine the association between dietary niacin intake and all-cause mortality as well as cardiovascular mortality. Three models were constructed for analysis. Model 1 was unadjusted. In Model 2, adjustments were made for age, race, and gender. Model 3 included adjustments for race, age, gender, education level, household income-to-poverty ratio, smoking status, alcohol consumption, body mass index (BMI), hypertension, diabetes, CKD, and HDL-C. Restricted cubic spline analysis was conducted to examine the nonlinear association between dietary niacin intake and mortality. Restricted spline analysis was performed using knot = 4 and the best model was selected based on the Akaike information criterion value. Further stratification was performed based on age, gender, education level, household income-to-poverty ratio, smoking status, alcohol consumption, and BMI. The significance of interaction was estimated using the p-value of the product term between dietary niacin intake and stratifying factors. Multiple imputation using chained equations method was employed for covariate imputation for variables with 15% missing data. All statistical analyses were conducted using R software (version 4.2.3), with a two-tailed *p* < 0.05 considered statistically significant.

## Results

### Baseline characteristics

This study included a total of 8744 diagnosed cases of metabolic syndrome, divided into four groups based on quartiles of dietary niacin intake, with baseline characteristics summarized in Table 1. Compared to participants with the lowest niacin intake, those with higher dietary niacin intake were more common among Non-Hispanic Whites, males, aged between 30 and 60, with higher BMI and higher education levels. There were no significant differences in cholesterol and triglycerides between the groups based on niacin intake.

**Table 1:** Baseline characteristics of participants with MetS in NHANES 2003 to 2018

Variables	Total (*n* = 8744)	Quartile 1 (*n* = 2156)	Quartile 2 (*n* = 2196)	Quartile 3 (*n* = 2203)	Quartile 4 (*n* = 2189)	*P*
Gender, n(%)						< 0.001
Male	4376 (50.05)	697 (32.33)	880 (40.07)	1197 (54.33)	1602 (73.18)	
Female	4368 (49.95)	1459 (67.67)	1316 (59.93)	1006 (45.67)	587 (26.82)	
Race, n(%)						< 0.001
Mexican	1544 (17.66)	438 (20.32)	376 (17.12)	370 (16.80)	360 (16.45)	
Hispanics	811 ( 9.27)	233 (10.81)	215 ( 9.79)	180 ( 8.17)	183 ( 8.36)	
Non-Hispanic White	4144 (47.39)	896 (41.56)	1051 (47.86)	1101 (49.98)	1096 (50.07)	
Non-Hispanic Black	1674 (19.14)	461 (21.38)	404 (18.40)	422 (19.16)	387 (17.68)	
Others	571 ( 6.53)	128 ( 5.94)	150 ( 6.83)	130 ( 5.90)	163 ( 7.45)	
Age, years, n(%)						< 0.001
<30	1574 (18 )	291 (13.50)	345 (15.71)	406 (18.43)	532 (24.30)	
30–60	3010 (34.42)	587 (27.23)	714 (32.51)	771 (35 )	938 (42.85)	
>60	4160 (47.58)	1278 (59.28)	1137 (51.78)	1026 (46.57)	719 (32.85)	
BMI, kg/m2, n(%)						< 0.001
<25	503 ( 5.75)	159 ( 7.37)	141 ( 6.42)	113 ( 5.13)	90 ( 4.11)	
25–30	2472 (28.27)	657 (30.47)	631 (28.73)	613 (27.83)	571 (26.08)	
>30	5769 (65.98)	1340 (62.15)	1424 (64.85)	1477 (67.04)	1528 (69.80)	
Education, n(%)						< 0.001
Less than high school	2650 (30.31)	902 (41.84)	667 (30.37)	594 (26.96)	487 (22.25)	
High school	2208 (25.25)	512 (23.75)	562 (25.59)	561 (25.47)	573 (26.18)	
Some college or above	3886 (44.44)	742 (34.42)	967 (44.03)	1048 (47.57)	1129 (51.58)	
Drinking status						< 0.001
Yes	5825 (66.62)	1204 (55.84)	1396 (63.57)	1568 (71.18)	1657 (75.70)	
No	2919 (33.38)	952 (44.16)	800 (36.43)	635 (28.82)	532 (24.30)	
Smoking status (%)						< 0.001
Yes	1495 (17.10)	375 (17.39)	364 (16.58)	379 (17.20)	377 (17.22)	
No	7249 (82.90)	1781 (82.61)	1832 (83.42)	1824 (82.80)	1812 (82.78)	
Diabetes, n(%)						< 0.001
Yes	2450 (28.02)	678 (31.45)	636 (28.96)	613 (27.83)	523 (23.89)	
No	6294 (71.98)	1478 (68.55)	1560 (71.04)	1590 (72.17)	1666 (76.11)	
Hypertension (%)						< 0.001
Yes	7268 (83.12)	1854 (85.99)	1808 (82.33)	1784 (80.98)	1822 (83.23)	
No	1476 (16.88)	302 (14.01)	388 (17.67)	419 (19.02)	367 (16.77)	
CVD, n(%)						< 0.001
Yes	1702 (19.46)	530 (24.58)	458 (20.86)	392 (17.79)	322 (14.71)	
No	7042 (80.54)	1626 (75.42)	1738 (79.14)	1811 (82.21)	1867 (85.29)	
CKD, n(%)						< 0.001
Yes	475 ( 5.43)	167 ( 7.75)	128 ( 5.83)	109 ( 4.95)	71 ( 3.24)	
No	8269 (94.57)	1989 (92.25)	2068 (94.17)	2094 (95.05)	2118 (96.76)	
HbA1c, %, M (Q₁, Q₃)	5.9 [5.5, 6.6]	6.0[5.5, 6.7]	5.9 [5.5, 6.6]	5.8 [5.5, 6.6]	5.800 [5.4, 6.4]	< 0.001
FBG, mg/dl, M (Q₁, Q₃)	114.0 [102.0, 133.0]	115.2[103.0, 136.0]	115.0 [102.0, 135.0]	114.0 [102.0, 132.0]	113.0 [101.0, 130.0]	0.0026
WC, cm, M (Q₁, Q₃)	109.5 [102.2, 119.4]	107.2 [99.8, 118.1]	109. 0 [100.8, 118.0]	109.7 [102.9, 119.2]	111.5 [104.3, 122.6]	< 0.001
HDL-C, mmol/L, M (Q₁, Q₃)	1.2 (1.0, 1.4)	1.1 (1.0, 1.4)	1.2 (1.0, 1.4)	1.2 (1.0, 1.4)	1.1 (1.0, 1.3)	0.236
LDL-C, mmol/L, M (Q₁, Q₃)	2.9 (2.3, 3.6)	2.9 (2.3, 3.6)	2.9 (2.3, 3.6)	2.9 (2.2, 3.6)	2.9 (2.3, 3.5)	0.761
TC, mmol/L, M (Q₁, Q₃)	5.0 (4.2, 5.7)	5.0 (4.2, 5.8)	5.0 (4.2, 5.7)	4.9 (4.2, 5.8)	5.0 (4.2, 5.7)	0.82
TG, mmol/L, M (Q₁, Q₃)	1.8 (1.3, 2.5)	1.8 (1.2, 2.3)	1.8 (1.3, 2.4)	1.9 (1.3, 2.5)	1.9 (1.3, 2.6)	< 0.001
AST, U/L, M (Q₁, Q₃)	23 [19, 29]	23 [19, 28]	23 [19, 27]	23 [19, 28 0]	25 [20, 30 0]	< 0.001
ALT, U/L, M (Q₁, Q₃)	23 [17, 32]	20 [15, 28 ]	22 [17, 29 ]	24 [18, 32]	27 [20, 37]	< 0.001
Creatine, mg/dl, M (Q₁, Q₃)	0.890 [0.730, 1.050]	0.850 [0.700, 1.050]	0.860 [0.720, 1.040]	0.900 [0.740, 1.060]	0.900 [0.770, 1.050]	< 0.001

Note: All percentages were weighted. Quartile1:0-15 mg/d, Quartile2: 15.1-20.1 mg/d, Quartile3: 21.2-28.6 mg/d. Quartile4: 28.7-143.3 mg/d. Abbreviation: MetS: metabolic syndrome; M: median; Q: quartile; Q1: 1st quartile; Q3: 3rd quartile; FPG: fasting plasma glucose; HbA1c: glycated hemoglobin; BMI: body mass index; WC: waist circumference; CVD: cardiovascular disease, CKD: chronic kidney disease; TG: triglyceride; HDL-C: high-density lipoprotein cholesterol; LDL-C: low-density lipoprotein cholesterol; TC: Total Cholesterol; AST: aspartate transaminase; ALT: glutamic-pyruvic transaminase

### Niacin Intake, all-cause and CVD mortality

**Fig. 2:**
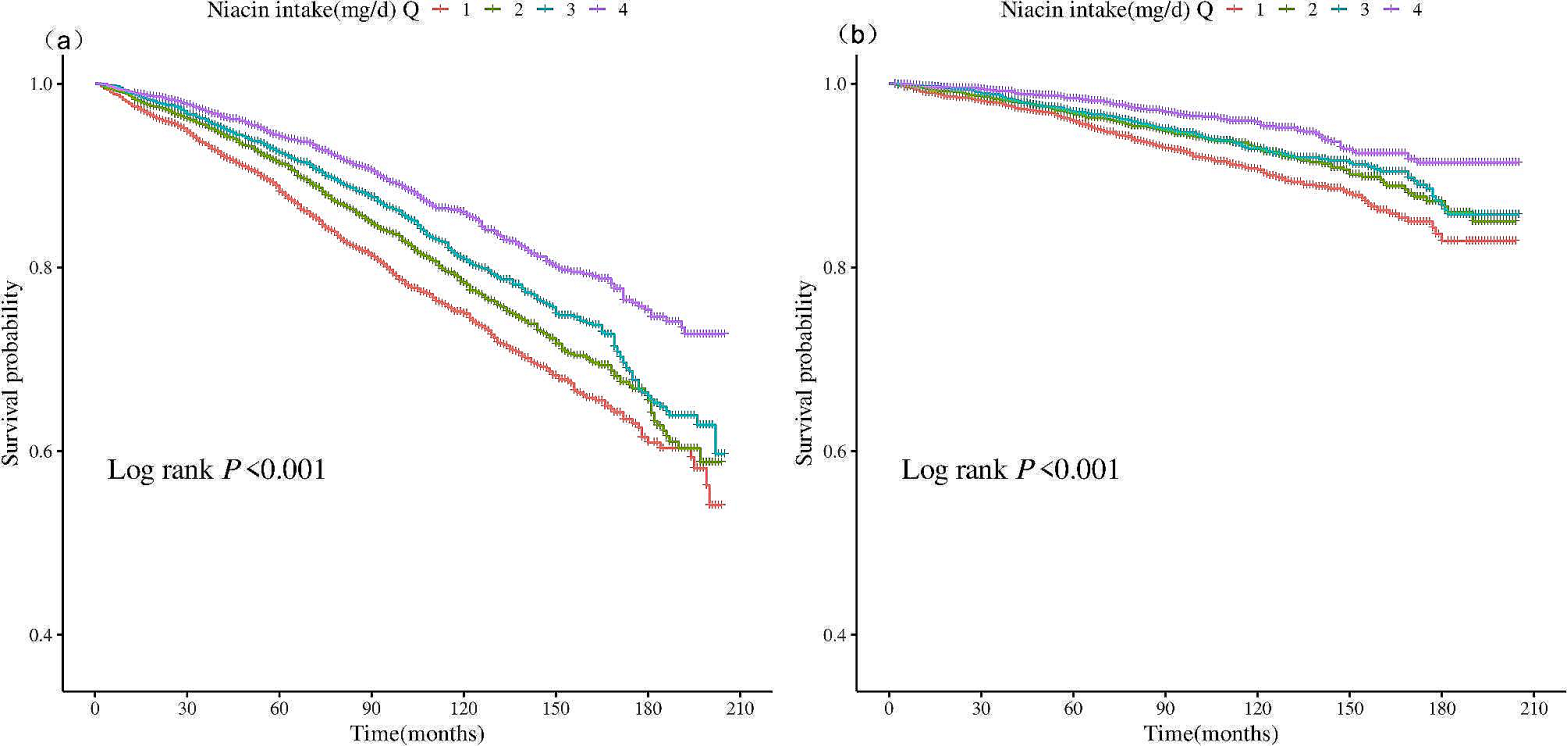
Kaplan-Meier mortality survival analysis curve

**Fig. 3:**
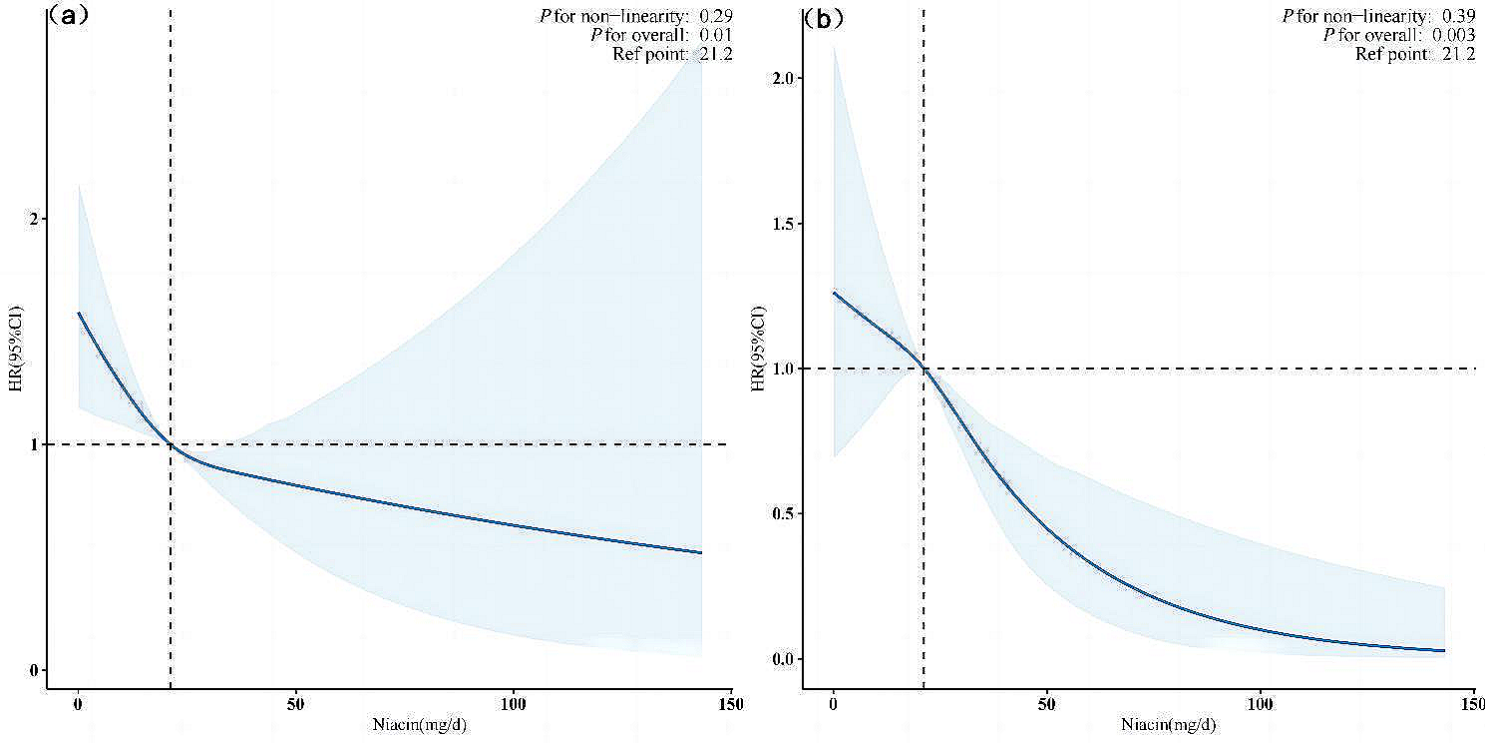
Restricted cubic spline models

During a median follow-up of 107 months, 1552 (17.7%) deaths were recorded, of which 511 (5.8%) were attributed to cardiovascular deaths. Kaplan-Meier analysis shown in Fig. 2 demonstrated significant differences in both all-cause and cardiovascular mortality risks among the different dietary niacin intake groups (log-rank *P* < 0.001), with a gradual reduction in all-cause and cardiovascular mortality risks as dietary niacin intake increased. As depicted in Table 2, in Model 1, compared to the reference group, the hazard ratio (HR) for all-cause mortality was 0.49 (95% CI, 0.38–0.62) (*P* < 0.001) for the fourth quartile, and the HR for cardiovascular mortality was 0.41 (95% CI, 0.28–0.61) (*P* < 0.001). After adjusting for age, sex, and race, in Model 2, the HR for all-cause mortality was 0.55 (95% CI, 0.39–0.78) (*P* < 0.001), and the HR for cardiovascular mortality was 0.62 (95% CI, 0.45–0.85) (*P* < 0.001). In the fully adjusted model, the HR for all-cause mortality for the fourth quartile was 0.68 (95% CI, 0.54–0.87) (*P* = 0.002), and the HR for cardiovascular mortality was 0.63 (95% CI, 0.39–0.78) (*P* < 0.001). The dose-response association between dietary niacin intake and both all-cause and CVD mortality rates is depicted in Fig. 3, where within the restricted cubic spline curves, there was a dose-response relationship between dietary niacin intake and all-cause mortality (*P* = 0.01) and CVD mortality (*P* = 0.02), with no significant nonlinear relationship observed (P for non-linearity > 0.05).

**Table 2:** The Cox regression analysis the association between the niacin intake and mortality of the population with MetS.

Subgroup	Model 1		Model 2		Model 3	
	HR (95% CI)	*P*	HR (95% CI)	*P*	HR (95% CI)	*P*
All-cause mortality						
Quartile1	1		1		1	
Quartile2	0.78(0.65, 0.95)*	0.015	0.79(0.66, 0.95)*	0.012	0.85(0.70, 1.02)	0.076
Quartile3	0.69(0.57, 0.84)**	< 0.001	0.67(0.55, 0.81)**	< 0.001	0.76(0.62, 0.94)*	0.010
Quartile4	0.49(0.38, 0.62)**	< 0.001	0.59(0.47, 0.74)**	< 0.001	0.68(0.54, 0.87)*	0.002
CVD mortality						
Quartile1	1		1		1	
Quartile2	0.68(0.50, 0.91)*	0.011	0.70(0.53, 0.93)*	0.013	0.75(0.53, 0.93)*	0.013
Quartile3	0.61(0.44, 0.85)*	0.004	0.62(0.45, 0.85)*	0.003	0.71(0.45, 0.85)*	0.003
Quartile4	0.41(0.28, 0.61)**	< 0.001	0.55(0.39, 0.78)**	< 0.001	0.63(0.39, 0.78)**	< 0.001

Note: *P<0.05, **P<0.001. Quartile1:0-15 mg/d, Quartile2: 15.1-20.1 mg/d, Quartile3: 21.2-28.6 mg/d. Quartile4: 28.7-143.3 mg/d. Abbreviation: MetS: metabolic syndrome. CVD: cardiovascular disease

Abbreviation: NHANES: National Health and Nutrition Examination Survey, MetS: metabolic syndrome

### Subgroup analyses

The subgroup analysis results were presented in Table S1 and Table S2, where no interaction was found with age, gender, BMI, education level, smoking, and alcohol consumption status (*P* for interaction > 0.05).

## Discussion

As the first study, the impact of niacin intake on long-term survival in the metabolic syndrome population, in this large-scale national cohort study involving American adults, it was found that higher niacin intake is associated with lower mortality risk among individuals with metabolic syndrome.

Despite being one of the earliest lipid-lowering drugs, the existence of the “niacin paradox” — the contradiction between niacin’s impact on lipid levels and its challenging achievement of the expected cardiovascular protective effects — has led to ongoing controversy regarding its cardiovascular effects [19]. Recent studies suggest the need to consider niacin’s adverse effects on the cardiovascular system. Based on current research, this study focused on individuals with metabolic syndrome and found a dose-response relationship between higher levels of niacin intake and decreased all-cause and cardiovascular mortality rates. This finding aligns with conclusions drawn from previous studies: increased niacin and B-complex vitamin intake can lower the incidence of metabolic syndrome [22]; individuals in the metabolic syndrome group have lower niacin levels [23, 24]; niacin treatment in patients with metabolic syndrome can improve HDL endothelial protective effects [25]; a clinical trial found that the 15-year total mortality rates for patients with metabolic syndrome receiving niacin and placebo treatments were 60% and 64%, respectively (risk ratio: 0.86) [26].

MetS incorporates risk factors for cardiovascular disease, and insulin resistance, oxidative stress, and chronic low-grade inflammation may play an important role in its pathophysiology [27]. According to the diagnostic criteria for metabolic syndrome, disturbances in carbohydrate and lipid metabolism play a more critical role. The trend of niacin-mediated normalization of mixed dyslipidemia associated with atherosclerosis, along with the alleviation of inflammation [28], improvement in insulin resistance status [29], and enhancement of endothelial function in a lipotoxic environment [30], may explain why niacin exerts a protective effect in individuals with metabolic syndrome.

The study provided some basis for the recommended amount of dietary niacin in the metabolic syndrome population, and indeed for the niacin paradox itself, there may be some dose-derived factors. According to the National Institutes of Health (NIH), the recommended dietary allowance (RDA) for niacin is 16 milligrams per day for adult males and 14 milligrams per day for adult females. In fact, nearly one-third of Americans do not meet this standard. In this study, the group with niacin intake exceeding 20.1 mg/day (the third and fourth quartiles) showed a significant reduction in all-cause and cardiovascular mortality risk compared to the reference group (the first quartile). Consideration of dose-related controversies in this study’s conclusions and previous research is warranted. It is noteworthy that in this high-quality meta-analysis, no cardiovascular protective effect of niacin was found. The niacin intake in the literature used as the control group ranged from 500 mg to 4000 mg per day [18], far exceeding the dietary intake in this study, and recent findings suggest that the pro-inflammatory effect of excess niacin metabolism can partially explain the niacin paradox [19]. The maximum daily niacin intake in this study was 143.3 mg, much lower than the daily dose of 2000 mg typically used in clinical randomized controlled trials [31]. Therefore, there may be a “U-shaped” relationship between niacin and cardiovascular effects - where risk increases after exceeding a certain threshold dose. This issue warrants further exploration in future research and requires a more comprehensive sample to elucidate the broader cardiovascular effects of niacin. This study was a nationwide and large-sample study. In conclusion, considering the current controversies surrounding niacin and its status as an easily supplemented essential nutrient, this study has made a certain contribution to the future health of the metabolic syndrome population.

### Limitations

Physical activity has been shown to affect the outcomes of metabolic syndrome [32]; however, due to incomplete physical activity data and the assumption that the missing values occurred randomly, this variable was not included in the study, potentially introducing some bias. Additionally, the niacin intake used in this study was based on 24-hour recall, which may be subject to recall bias and selective reporting. Furthermore, niacin intake is also associated with supplemental niacin use; however, since niacin intake as a nutritional supplement was not reported in NHANES before 2005–2006, this important variable was not included, posing another source of bias in this study. Nevertheless, the population consuming niacin dietary supplements is relatively small, hence the impact on the results is considered to be minimal.

## Conclusion

This cohort study, conducted using nationally representative data, suggests that increasing niacin intake may reduce the risk of mortality, providing guidance for dietary niacin intake in this population.

### Electronic supplementary material

Below is the link to the electronic supplementary material.

Supplementary Material 1

## Data Availability

No datasets were generated or analysed during the current study.

## Abbreviations

MetS: Metabolic syndrome
CVD: Cardiovascular disease
NHANES: National Health and Nutrition Examination Survey
WWEIA: What We Eat in America
USDA: United States Department of Agriculture
DHHS: Department of Health and Human Services
MEC: Mobile Examination Center
NCEP ATP III: National Cholesterol Education Program Adult Treatment Panel III
FBG: Fasting blood glucose
HDL-C: High-density lipoprotein cholesterol
TG: Triglycerides
BMI: Body mass index
WC: Waist circumference
LDL-C: Low-density lipoprotein cholesterol
TC: Total Cholesterol
ALT: Alanine aminotransferase
AST: Aspartate aminotransferase
NDI: National Death Index
NCHS: National Center for Health Statistics
ICD-10: International Classification of Diseases 10th Revision
HR: Hazard ratio
CI: Confidence interval
CKD: Chronic kidney disease
NIH: National Institutes of Health
RDA: Recommended dietary allowance
