# Supplementary material for: Dietary niacin Intake and its association with all-cause and cardiovascular mortality rates in individuals with metabolic syndrome
Source: Nutr J. 2024 Aug 10;23:90. doi: 10.1186/s12937-024-00993-7 (PMC11316429; doi:10.1186/s12937-024-00993-7)
Supplement: Supplementary file 1 — Supplementary Material 1 [file 12937_2024_993_MOESM1_ESM.docx]

**Dietary Niacin Intake and Its Association with All-Cause and Cardiovascular Mortality Rates in Individuals with Metabolic Syndrome**

Yuqing Fu^1^, CongXu^1^, and Guifu Wu^1*^

**Author Affiliation**

^1^ Department of Cardiology, The Eighth Affiliated Hospital of Sun Yat-sen University

, Shenzhen , 518000, Guangdong, China

^*^ Corresponding Author: Guifu Wu

**Table S1. Subgroup analysis of all-cause mortality among MetS population.**

| Subgroup | Niacin(mg/d) | HR(95%CI) | *P* value | *P* for interaction |
| --- | --- | --- | --- | --- |
| Gender |  |  |  | 0.43 |
| Male |  |  |  |  |
|  | Quartile1 | 1 |  |  |
|  | Quartile2 | 0.93(0.73,1.18) | 0.53 |  |
|  | Quartile3 | 0.79(0.61,1.01) | 0.06 |  |
|  | Quartile4 | 0.69(0.51,0.93)* | 0.02 |  |
|  | Trend test |  | 0.01 |  |
| Female |  |  |  |  |
|  | Quartile1 | 1 |  |  |
|  | Quartile2 | 0.8(0.63,1.03) | 0.08 |  |
|  | Quartile3 | 0.72(0.53,0.99)* | 0.04 |  |
|  | Quartile4 | 0.85(0.57,1.26) | 0.42 |  |
| Trend test |  |  | 0.14 |  |
| Age,years |  |  |  | 0.79 |
| ＜40 |  |  |  |  |
|  | Quartile1 | 1 |  |  |
|  | Quartile2 | 1.05(0.28,3.86) | 0.95 |  |
|  | Quartile3 | 1.65(0.47,5.77) | 0.43 |  |
|  | Quartile4 | 0.55(0.1,2.93) | 0.48 |  |
|  | Trend test |  | 0.45 |  |
| 40-60 |  |  |  |  |
|  | Quartile1 | 1 |  |  |
|  | Quartile2 | 1.02(0.64,1.61) | 0.95 |  |
|  | Quartile3 | 1.02(0.62,1.69) | 0.92 |  |
|  | Quartile4 | 0.99(0.6,1.64) | 0.97 |  |
|  | Trend test |  | 0.96 |  |
| ＞60 |  |  |  |  |
|  | Quartile1 | 1 |  |  |
|  | Quartile2 | 0.85(0.7,1.03) | 0.09 |  |
|  | Quartile3 | 0.7(0.57,0.86)* | <0.001 |  |
|  | Quartile4 | 0.64(0.51,0.81)* | <0.001 |  |
| Trend test |  |  | <0.001 |  |
| BMI |  |  |  | 0.256 |
| ＜25 |  |  |  |  |
|  | Quartile1 | 1 |  |  |
|  | Quartile2 | 0.71(0.39,1.29) | 0.26 |  |
|  | Quartile3 | 0.57(0.27,1.2) | 0.14 |  |
|  | Quartile4 | 0.7(0.35,1.4) | 0.31 |  |
| Trend test |  |  | 0.14 |  |
| 25-30 |  |  |  |  |
|  | Quartile1 | 1 |  |  |
|  | Quartile2 | 1.03(0.75,1.43) | 0.84 |  |
|  | Quartile3 | 0.62(0.43,0.88)* | 0.01 |  |
|  | Quartile4 | 0.84(0.57,1.25) | 0.39 |  |
|  | Trend test |  | 0.08 |  |
| ＞30 |  |  |  |  |
|  | Quartile1 | 1 |  |  |
|  | Quartile2 | 0.83(0.64,1.07) | 0.16 |  |
|  | Quartile3 | 0.92(0.69,1.22) | 0.55 |  |
|  | Quartile4 | 0.66(0.48,0.92)* | 0.01 |  |
|  | Trend test |  | 0.04 |  |
| Educational level |  |  |  | 0.091 |
| <High school |  |  |  |  |
|  | Quartile1 | 1 |  |  |
|  | Quartile2 | 1.1(0.85,1.43) | 0.48 |  |
|  | Quartile3 | 0.89(0.65,1.23) | 0.5 |  |
|  | Quartile4 | 0.92(0.64,1.34) | 0.67 |  |
|  | Trend test |  | 0.43 |  |
| High school |  |  |  |  |
|  | Quartile1 | 1 |  |  |
|  | Quartile2 | 0.83(0.6,1.14) | 0.24 |  |
|  | Quartile3 | 0.66(0.45,0.96)* | 0.03 |  |
|  | Quartile4 | 0.79(0.5,1.25) | 0.32 |  |
|  | Trend test |  | 0.21 |  |
| Some college or above |  |  |  |  |
|  | Quartile1 | 1 |  |  |
|  | Quartile2 | 0.67(0.49,0.92)* | 0.01 |  |
|  | Quartile3 | 0.67(0.47,0.96)* | 0.03 |  |
|  | Quartile4 | 0.52(0.35,0.76)* | <0.001 |  |
|  | Trend test |  | <0.001 |  |
| Drinking status |  |  |  | 0.198 |
| Yes |  |  |  |  |
|  | Quartile1 | 1 |  |  |
|  | Quartile2 | 0.86(0.68,1.08) | 0.2 |  |
|  | Quartile3 | 0.77(0.59,1) | 0.05 |  |
|  | Quartile4 | 0.62(0.46,0.85)* | 0.003 |  |
|  | Trend test |  | 0.002 |  |
| No |  |  |  |  |
|  | Quartile1 | 1 |  |  |
|  | Quartile2 | 0.88(0.68,1.15) | 0.35 |  |
|  | Quartile3 | 0.79(0.54,1.16) | 0.22 |  |
|  | Quartile4 | 0.97(0.67,1.41) | 0.88 |  |
|  | Trend test |  | 0.61 |  |
| Smoking status |  |  |  | 0.658 |
| Yes |  |  |  |  |
|  | Quartile1 | 1 |  |  |
|  | Quartile2 | 0.88(0.57,1.36) | 0.55 |  |
|  | Quartile3 | 0.88(0.55,1.39) | 0.58 |  |
|  | Quartile4 | 0.72(0.43,1.21) | 0.22 |  |
|  | Trend test |  | 0.23 |  |
| No |  |  |  |  |
|  | Quartile1 | 1 |  |  |
|  | Quartile2 | 0.87(0.72,1.05) | 0.14 |  |
|  | Quartile3 | 0.75(0.61,0.91)* | 0.004 |  |
|  | Quartile4 | 0.73(0.56,0.95)* | 0.02 |  |
|  | Trend test |  | 0.01 |  |
| Family income to poverty ratio |  |  |  | 0.601 |
| <1.0 |  |  |  |  |
|  | Quartile1 | 1 |  |  |
|  | Quartile2 | 0.94(0.67,1.32) | 0.71 |  |
|  | Quartile3 | 0.9(0.62,1.31) | 0.58 |  |
|  | Quartile4 | 1.02(0.64,1.62) | 0.95 |  |
|  | Trend test |  | 0.94 |  |
| 1.0-3.0 |  |  |  |  |
|  | Quartile1 | 1 |  |  |
|  | Quartile2 | 0.85(0.66,1.11) | 0.23 |  |
|  | Quartile3 | 0.67(0.51,0.88)* | 0.004 |  |
|  | Quartile4 | 0.62(0.44,0.86)* | 0.005 |  |
|  | Trend test |  | <0.001 |  |
| >3.0 |  |  |  |  |
|  | Quartile1 | 1 |  |  |
|  | Quartile2 | 0.73(0.51,1.04) | 0.08 |  |
|  | Quartile3 | 0.77(0.51,1.16) | 0.21 |  |
|  | Quartile4 | 0.68(0.45,1.04) | 0.07 |  |

Note: *P＜0.05, MetS: metabolic syndrome. Quartile1:0-15mg/d, Quartile2: 15.1-20.1mg/d, Quartile3: 21.2-28.6mg/d. Quartile4: 28.7-143.3mg/d.

**Table S2. Subgroup analysis of CVD mortality among MetS population.**

| Subgroup | Niacin(mg/d) | HR(95%CI) | P value | P for interaction |
| --- | --- | --- | --- | --- |
| Gender |  |  |  | 0.276 |
| Male |  |  |  |  |
|  | Quartile1 | 1 |  |  |
|  | Quartile2 | 0.98（0.67,1.44) | 0.93 |  |
|  | Quartile3 | 0.93（0.58,1.49) | 0.77 |  |
|  | Quartile4 | 0.72（0.45,1.17) | 0.19 |  |
|  | Trend test |  | 0.17 |  |
| Female |  |  |  |  |
|  | Quartile1 | 1 |  |  |
|  | Quartile2 | 0.67（0.43,1.02) | 0.06 |  |
|  | Quartile3 | 0.58（0.35,0.95)* | 0.03 |  |
|  | Quartile4 | 0.8（0.47,1.34) | 0.39 |  |
| Trend test |  |  | 0.11 |  |
| Age,years |  |  |  | 0.309 |
| ＜40 |  |  |  |  |
|  | Quartile1 | 1 |  |  |
|  | Quartile2 | 0.68（0.13,3.67) | 0.66 |  |
|  | Quartile3 | 0.09（0.01,0.8)* | 0.03 |  |
|  | Quartile4 | 0.41（0.13,1.35) | 0.14 |  |
|  | Trend test |  | 0.08 |  |
| 40-60 |  |  |  |  |
|  | Quartile1 | 1 |  |  |
|  | Quartile2 | 0.99（0.45,2.16) | 0.98 |  |
|  | Quartile3 | 0.59（0.25,1.36) | 0.22 |  |
|  | Quartile4 | 0.66（0.26,1.68) | 0.38 |  |
|  | Trend test |  | 0.3 |  |
| ＞60 |  |  |  |  |
|  | Quartile1 | 1 |  |  |
|  | Quartile2 | 0.74（0.55,1 | 0.05 |  |
|  | Quartile3 | 0.79（0.55,1.15) | 0.22 |  |
|  | Quartile4 | 0.69（0.47,1.02) | 0.06 |  |
| Trend test |  |  | 0.08 |  |
| BMI |  |  |  | 0.394 |
| ＜25 |  |  |  |  |
|  | Quartile1 | 1 |  |  |
|  | Quartile2 | 0.96（0.31,2.99) | 0.94 |  |
|  | Quartile3 | 0.5（0.14,1.73) | 0.27 |  |
|  | Quartile4 | 1.21（0.41,3.54) | 0.73 |  |
| Trend test |  |  | 0.94 |  |
| 25-30 |  |  |  |  |
|  | Quartile1 | 1 |  |  |
|  | Quartile2 | 0.65（0.34,1.25) | 0.2 |  |
|  | Quartile3 | 0.7（0.37,1.34) | 0.29 |  |
|  | Quartile4 | 0.48（0.23,1.02) | 0.05 |  |
|  | Trend test |  | 0.07 |  |
| ＞30 |  |  |  |  |
|  | Quartile1 | 1 |  |  |
|  | Quartile2 | 0.81（0.55,1.18) | 0.28 |  |
|  | Quartile3 | 0.78（0.49,1.24) | 0.3 |  |
|  | Quartile4 | 0.66（0.38,1.16) | 0.15 |  |
|  | Trend test |  | 0.17 |  |
| Educational level |  |  |  | 0.109 |
| <High school |  |  |  |  |
|  | Quartile1 | 1 |  |  |
|  | Quartile2 | 0.89（0.58,1.39) | 0.62 |  |
|  | Quartile3 | 1.23（0.82,1.85) | 0.32 |  |
|  | Quartile4 | 1.04（0.57,1.87) | 0.91 |  |
|  | Trend test |  | 0.54 |  |
| High school |  |  |  |  |
|  | Quartile1 | 1 |  |  |
|  | Quartile2 | 0.73（0.45,1.18) | 0.2 |  |
|  | Quartile3 | 0.62（0.31,1.25) | 0.18 |  |
|  | Quartile4 | 0.74（0.38,1.41 | 0.36 |  |
|  | Trend test |  | 0.34 |  |
| Some college or above |  |  |  |  |
|  | Quartile1 | 1 |  |  |
|  | Quartile2 | 0.61（0.37,1.01 | 0.06 |  |
|  | Quartile3 | 0.49（0.25,0.94)* | 0.03 |  |
|  | Quartile4 | 0.4（0.21,0.77)* | 0.01 |  |
|  | Trend test |  | 0.01 |  |
| Drinking status |  |  |  | 0.823 |
| Yes |  |  |  |  |
|  | Quartile1 | 1 |  |  |
|  | Quartile2 | 0.71（0.48,1.07) | 0.1 |  |
|  | Quartile3 | 0.63（0.43,0.94)* | 0.02 |  |
|  | Quartile4 | 0.56（0.35,0.9)* | 0.02 |  |
|  | Trend test |  | 0.02 |  |
| No |  |  |  |  |
|  | Quartile1 | 1 |  |  |
|  | Quartile2 | 0.87（0.55,1.39) | 0.57 |  |
|  | Quartile3 | 0.95（0.5,1.83) | 0.89 |  |
|  | Quartile4 | 0.88（0.47,1.64) | 0.69 |  |
|  | Trend test |  | 0.75 |  |
| Smoking status |  |  |  | 0.492 |
| Yes |  |  |  |  |
|  | Quartile1 | 1 |  |  |
|  | Quartile2 | 0.83（0.4,1.75) | 0.63 |  |
|  | Quartile3 | 0.5（0.24,1.03) | 0.06 |  |
|  | Quartile4 | 0.27（0.1,0.73)* | 0.01 |  |
|  | Trend test |  | 0.003 |  |
| No |  |  |  |  |
|  | Quartile1 | 1 |  |  |
|  | Quartile2 | 0.79（0.57,1.09) | 0.15 |  |
|  | Quartile3 | 0.8（0.54,1.17) | 0.25 |  |
|  | Quartile4 | 0.81（0.53,1.23) | 0.32 |  |
|  | Trend test |  | 0.37 |  |
| Family income to poverty ratio |  |  |  | 0.89 |
| <1.0 |  |  |  |  |
|  | Quartile1 | 1 |  |  |
|  | Quartile2 | 0.9（0.54,1.5) | 0.7 |  |
|  | Quartile3 | 1.05（0.61,1.8) | 0.86 |  |
|  | Quartile4 | 0.64（0.24,1.74) | 0.38 |  |
|  | Trend test |  | 0.47 |  |
| 1.0-3.0 |  |  |  |  |
|  | Quartile1 | 1 |  |  |
|  | Quartile2 | 0.69（0.46,1.06) | 0.09 |  |
|  | Quartile3 | 0.64（0.39,1.03) | 0.07 |  |
|  | Quartile4 | 0.54（0.33,0.9)* | 0.02 |  |
|  | Trend test |  | 0.01 |  |
| >3.0 |  |  |  |  |
|  | Quartile1 | 1 |  |  |
|  | Quartile2 | 0.68（0.35,1.31 | 0.25 |  |
|  | Quartile3 | 0.64（0.32,1.31 | 0.22 |  |
|  | Quartile4 | 0.62（0.33,1.16) | 0.14 |  |

Note: *P＜0.05, MetS: metabolic syndrome. Quartile1:0-15mg/d, Quartile2: 15.1-20.1mg/d, Quartile3: 21.2-28.6mg/d. Quartile4: 28.7-143.3mg/d.
